# Student intrinsic motivational states underlie cognitive performance regardless of academic stage

**DOI:** 10.3389/fpsyg.2026.1896397

**Published:** 2026-08-26

**Authors:** Simon Hanzal, Enya Kelly, Hannah Baughan

**Affiliations:** School of Psychology and Neuroscience, University of Glasgow, Glasgow, United Kingdom

**Keywords:** motivation, motivational state, performance, self-determination theory, sustained attention, working memory

## Abstract

Motivation has been identified as an important factor affecting working memory performance. Notably, different groups such as older individuals are known to successfully compensate for a deficiency in processing speed through increased intrinsic motivation to perform well. However, the extent to which these motivational effects generalize to the student sample commonly employed in the study of working memory remains unclear. We tested the relationship of motivation to performance on a 2-back working memory task, while manipulating baseline motivation levels through recruitment of student groups with prospectively diverse motivational drivers (credited first years vs. paid final years) and captured their perceived intrinsic motivation. Motivation was also assessed behaviourally through participants' free choice to complete additional unrewarded task blocks. Participants reporting higher motivation showed faster and more efficient responses, but were not motivated to spend more time voluntarily engaging with the task. Furthermore, we found that neither the difference in academic year (first year vs. final year) nor an overall sense of meaning (operationalised as eudaimonic wellbeing) was associated with student performance. Overall, the findings help to outline the degree of influence of intrinsic motivation on working memory performance. More specifically, we highlight a gap between clear performance supported through self-reported intrinsic motivation and more objective markers of motivation. Given the emerging societal and educational shifts and pressures within the United Kingdom, the findings provide implications for higher educational contexts in developing strategies to understand students' IM during university and ultimately enhance their performance.

## 1 Introduction

The relationship of motivational states to task performance has attracted widespread interest in psychological research (Braver et al., 2014; Bandhu et al., 2024). Simply described, motivation is a movement toward an action (Deci and Ryan, 2008). As such, it is considered a driver or at least a decisive factor in improved task performance across a range of settings (Botvinick and Braver, 2015).

Motivation can be studied through motivational states, understood as self-reported fluctuations in experienced motivation during a task. Motivational states lend themselves to an easy investigation in the laboratory (Botvinick and Braver, 2015) with a measurable impact during typical laboratory tasks (Swirsky et al., 2023). There, the entrainment of performance and motivation can be tracked across the task trajectory (Roe and Inceoglu, 2016; Reteig et al., 2019) or compared in different reward conditions (Robison et al., 2021; Swirsky et al., 2023). Of these two approaches, motivational states were more commonly observed to impact performance through changes made to the reward value of the undertaken task through reward manipulations (Braver et al., 2014). Researchers would introduce an incentive or alter task instructions to change the reward context of the task, commonly through monetary incentives (Engelmann et al., 2009; Swirsky et al., 2023). For instance, introducing unexpected financial rewards mid-task reduced subjective mental fatigue and boosted both physiological engagement and objective task performance in a working memory task (Wójcik and Necka, 2024). Similarly, performance-contingent rewards improved both motivation and performance on a maths and language test (Hendijani et al., 2016). Overall, there is an extensive body of literature demonstrating a consistent link between motivation, incentives and performance (Cerasoli et al., 2014; Howard et al., 2021).

Generally, such shifts in motivational states are often coupled with long-term dispositional traits which affect their dynamics, as seen in the varied theoretical conceptualisations of motivation (Braver et al., 2014; Bandhu et al., 2024). These are sometimes framed as trait motivation and commonly reported by participants as an experience across a longer period preceding the task (Locke and Braver, 2008; Roe and Inceoglu, 2016). The relation of trait to state motivation remains consistent with the overall account of motivation as a director of arousal and persistence of behavior, working across different timescales (Roe and Inceoglu, 2016), as widely seen in literature (Choi et al., 2012; Wasserman and Wasserman, 2020; Faust, 2021). However, the understanding of trait motivation varies significantly more than that of motivational states and is actually often seen as a complex array of inter-related factors (Deci and Ryan, 2008; Bandhu et al., 2024).

Among different conceptualisations of trait motivation, a widely employed perspective on motivation distinguishes between extrinsic motivation (EM) and intrinsic motivation (IM) (Ryan et al., 1983; Braver et al., 2014). IM refers to a drive to engage in an activity for one's own personal interest and satisfaction, with an absence of external pressures or rewards. Conversely, EM is oriented toward the activity's external outcomes (Ryan and Deci, 2000). Under this account, improved motivational states arise from an inherent interest and pleasure associated with the task (Choi et al., 2012) or fluctuate and drop along with an added onset of fatigue (Hopstaken et al., 2015; Kok, 2022) because of an inherently boring or unsatisfying nature of the task (Herlambang et al., 2021). Higher motivational states may be attributed to IM more specifically when tasks align to specific central and internal goals of individuals. The Means-Ends Fusion Model (Kruglanski et al., 2018) further expands on this, postulating that activities where the task and the goal are the same encourage the highest IM to perform. In other words, pursuing an activity becomes rewarding when the goal gained cannot be distinguished from the activity itself (Fishbach and Woolley, 2022). It thus follows that IM is best captured in studies where the reward value of the task remains constant. Conversely, paradigms employing manipulations of reward value would focus on the extrinsic aspect of motivation.

IM has previously been observed to underlie behavioral effects. This is seen in a study by Bieg et al. (2017), where students who studied with the goal of mastering information, rather than only to perform well in a test, reported being more intrinsically motivated. Froiland and Worrell (2016) revealed that IM improved high school academic performance through goals relating to learning and academic achievement. Workplace studies showed similar findings, where motivation related to career goals led to higher reported job performance and productivity (Hogenelst et al., 2022; Roussillon Soyer et al., 2022). As seen above, strong reliance on mainly self-report measures is very common when studying IM (Isiksal, 2010; Augustyniak et al., 2016; Almulla et al., 2025). However, this blurs how motivational states, as impacted by long-term IM, directly lead to altered performance. A predominantly subjective report-based approach has previously been criticized for the limited insight into dynamic motivational processes (Roe and Inceoglu, 2016) and for introducing response bias (Fryer and Dinsmore, 2020).

Some efforts were made to map the influence of IM on objective performance. Notably, in a study of sustained attention performance, Robison et al. (2021) showed that specific goals tied to IM offset vigilance decrements, illustrating a potential impact of IM. This is paralleled by Ramme et al. (2022), who employed a visual modality-adaptive n-back and a parametric go/nogo task (PGNG) to examine motivational differences among participants, using four items from the IM Inventory (IMI) and aiming to track the relationship of IM to performance. The different styles of tasks introduced varying levels of IM and higher IM was associated with higher ability on the task.

Similarly to Ramme et al. (2022); Hanzal et al. (2025) have manipulated baseline IM levels, achieving this by targeting two different samples: a sample of university students (mean age = 19) and of healthy older adults (mean age = 69.5, screened for dementia). Effects of motivation were seen both in the baseline performance of the groups, but also in a different response to a reward incentive. Students were shown to be more prone to manipulations of their motivational levels, with stronger entrained performance gains. Higher baseline IM instead explained strong performance in older adults prior to the reward, despite an expected onset of aging-related decline (Murman, 2015). This was attributed to age influences on baseline levels of IM in experimental participants (Swirsky et al., 2023). Along with this reasoning, the authors likewise argued that higher IM in older adults allowed them to compensate for the decline through maintained or better performance. The effects observed are partly owed to an arguably different proclivity of the two chosen populations to react to explicit manipulations of motivation (Swirsky et al., 2023), reflecting different levels of IM during cognitive tasks. As an interim conclusion, we understood that relying on populations differing in their baseline motivation can elicit differences in baseline motivation and thus show direct effects on performance. However, sampling from different age groups means manipulating baseline motivation levels along with introducing other, notably uncontrollable confounds. These center around biological differences due to aging (Carriere et al., 2013; Murman, 2015), with generational perceptions unlikely to explain this effect (Costanza et al., 2023).

Hence, the present study refines the former performance-based paradigms to examine how motivation influences performance in an age-matched group of students by attempting to introduce variable baseline motivational levels and sampling from different year groups, but within the same young student population. To achieve this, we selected the n-back as a task previously highlighted as the most sensitive to dynamic shifts in motivational state and populational-level difference (Swirsky et al., 2023). The n-back has been noted to capture sustained attention similarly to the SART (Barrington and Miller, 2023). Moreover, with additional relevance to motivational drivers, the n-back requires active maintenance, updating, decision-making, and cognitive flexibility. This was best seen in Swirsky et al. (2023), where n-back paradigms elicited a strong age-related motivational effect. The n-back likewise contains a strong sustained attention component as a repetitive and effortful task, where executive functions are vulnerable to lapses and vigilance decrements; therefore, motivation can be posited as a central variable for sustaining engagement and performance (Warm et al., 2008; Engelmann et al., 2009; Reteig et al., 2019).

In contrast with the previous reward paradigm (Hanzal et al., 2025), the current study excludes performance-contingent rewards and feedback, enabling an estimation of how individuals' baseline IM state directly maps onto task performance (Satterthwaite et al., 2012). This follows more closely the reasoning of Ryan and Deci (2000), outlining the need to consider IM as manifested under specific conditions, including contexts without an incentive. Students are understood as possibly inhibited in their IM state (DeRight and Jorgensen, 2015), and were seen as such in our past work (Hanzal et al., 2025). However, the student population as a whole varies in the distribution of motivational levels, with past work reporting both undermotivated and motivated subgroups (Dunn et al., 2019). In this way, intentional sampling of students may more easily allow for demonstrating a direct relationship between IM and performance without a reliance on a reward paradigm. Selection of varied cohorts among students may thus serve as a similar between-subject manipulation of motivational levels inspired by our past work, without introducing performance-related confounds stemming from aging effects (Swirsky et al., 2023).

In light of this, we note that IM differs between early and late stages of undergraduate study (D'Lima et al., 2014; Corpus et al., 2020; Roozen et al., 2024). The first and final years of university represent critical transition points in the academic trajectory and are therefore informative stages for examining motivational differences. First-year students enter university with heightened anticipation and curiosity, alongside uncertainty and apprehension as they adjust to unfamiliar academic and social environments (Beckett, 2024; Osafo et al., 2025). They are typically exposed to experimental participation as a requirement of their course, serving as an opportunity to improve their learning (Rosell et al., 2005; Flynn and Rocheleau, 2024). While final-year students face intensified pressure from exams, degree classification and future employment (Keane et al., 2021; Yang et al., 2025). As an example, Brouse et al. (2010) revealed that freshmen reported significantly higher IM and EM than seniors, indicating a decline in motivation. This finding is grounded in the notion proposed by Ryan and Deci (2000) that as students progress through their academic journey, they become less self-determined, decreasing their IM. Moreover, recent research provides some evidence that IM as an independent predictor declines from first to fourth year, attributable to academic demands, burnout, career-related pressures and diminishing novelty of university life (Blindheim et al., 2025). Motivation appears to function differently across the stages of undergraduate study, with distinct effects on performance. Comparing first- and final-year students therefore offers a novel between-subject manipulation of IM in students to aid testing its relationship with cognitive performance.

Classical theories of IM understand there to be a specific set of goals and underlying motivators that contribute to the onset of IM states during a task (Ryan and Deci, 2000). As conceptualized through SDT, eudaimonic wellbeing goals are strongly associated with subjective report of IM (Waterman and Schwartz, 2024). Most notably, this has been mapped in the older sample by Ryan and Campbell (2021), showing a stronger social, rather than financial orientation. This reasoning supports the notion that orientation toward mastery and personal growth may be a driver of these differences. When autonomy and competence are emphasized in behavior, SDT posits a notable growth both in IM and wellbeing (Bandhu et al., 2024). Eudaimonic wellbeing, which encompasses personal growth, achieving goals and a sense of meaning in life, is generally understudied within motivation and general wellbeing research (Su et al., 2020). Peiró et al. (2019) measured eudaimonic and hedonic wellbeing of employees twice daily over 4 days, and compared the results with their supervisors' evaluation of their work performance. The results showed that activities which were perceived as worthwhile produced higher ratings of work performance. Likewise, Kożusznik et al. (2019) found that eudaimonic wellbeing fluctuates daily which accounts for workplace performance shifts. Interestingly, Waterman et al. (2003) found that eudaimonic identity, relating to fulfillment and personal expressiveness, is strongly linked to both wellbeing and motivation in a work context. Taking this evidence all together, IM may be rooted in eudaimonic wellbeing and its associated goals. A eudaimonic wellbeing construct seems thus well suited to further investigate underlying drivers of motivation reported during the task and was included in this study.

In addition to the main experimental task, this study incorporates a free-choice measure (FCM) (Deci, 1971) as an experimental measure of underlying motivation. Voluntarily spent time is theorized to reflect individuals' IM, cognitive engagement, and desire to improve performance or mastery (Deci and Ryan, 1985; Guay et al., 2000). Traditionally, the FCM demonstrates an “undermining effect,” whereby the removal of external rewards typically decreases IM and task engagement (Deci, 1971; Deci et al., 1999). In contrast, this study uses the paradigm to provide an additional motivational metric related to voluntary engagement on a cognitive task, without manipulating reward removal and in a context where external incentives are not contingent on performance. Incorporating this more overt measure of motivation strengthens the study by adding another behavioral dimension and prospectively rooting the present investigation in SDT conceptualisations of IM.

Motivational states underlie cognitive performance, particularly in experimental paradigms utilizing working memory and sustained attention. These motivational states may be rooted in baseline differences in IM which can be manipulated to lead to different levels of performance. Through targeted sampling of students in their first- and final-years, it may be possible to undertake a between-subject manipulation of IM, as was previously seen in an approach using different age groups, and without associated aging-related confounds. The present study thus employed a converging performance-based paradigm to directly assess the connection between performance on the n-back and IM captured through the IMI to address the following hypotheses: It is hypothesized that students with higher IM will achieve better performance on the n-back task. Additionally, given the evidence that motivation fluctuates across the academic trajectory, it is further hypothesized that the relationship between IM and task performance will differ between first- and final-year undergraduate students. Finally, it was predicted that students with higher eudaimonic wellbeing will show higher IM.

## 2 Materials and methods

### 2.1 Participants

The experimental design and hypotheses were pre-registered on the Open Science Framework (https://osf.io/pyzn7). By utilizing effect size parameters from previous research in the field of IM and performance (Robinson et al., 2012), a power analysis was performed to determine the required sample size for this study. It revealed that to maintain a power of 0.8 (Cohen, 1992) at a significance of 0.05, and assuming a medium effect size (*r* = 0.431), a sample size of 40 was required. Power calculations were conducted using the ‘pwr' R package (Version 4.5.2, R Core Team, 2025) to determine the required sample size to detect this effect with a two-sided hypothesis test. Although the study was initially preregistered to achieve 90% power, recruitment constraints led us to revise this target to the conventional 80% (Cohen, 1992) before data collection began.

Thirty-nine participants took part in the study between 19/11/2025 and 02/02/2026. Participants were recruited from the first- and final-year (fourth) psychology student cohorts from the University of Glasgow (age >17) via the university's participant pool and on-campus advertisements. First-year students received course credits, while final-year students received £10 vouchers. All participants reported no medical conditions or visual impairments that could affect cognitive task performance. No participants reported epilepsy or less than 5 h of sleep (see Dinges et al., 1997) and all fully completed the practice, main n-back task and the IMI. Data was scanned for poor task performance. One participant failed to meet the minimum required standard for the task (>75% accuracy) in the practice trials and was excluded from analysis. IMI data were scanned for careless responses using the “careless” package (v1.2.2; Yentes and Wilhelm, 2018), and outliers were identified using boxplots. Three participants were identified as potential cases of straight lining; however, none reached thresholds of extreme inattentive responding, and their performance accuracy remained above chance, therefore they were retained.

The final sample of the study consisted of 38 participants, yielding a power of 0.79. Participants comprised 19 first-year students (6 male, 10 female, 3 non-binary, *M*_age_ = 18.53*, SD* = 0.61, righthanded = 16, lefthanded = 3, ambidextrous = 0) and 19 final-year students (5 male, 14 female, 0 non-binary, *M*_age_ = 22.21*, SD* = 3.94, righthanded = 18, lefthanded = 0, ambidextrous = 1). The study adhered to the British Psychological Society code of human research ethics (British Psychological Society, 2014) and received ethical approval from the University of Glasgow College of Medical Veterinary and Life Sciences (approval number: 200230387).

### 2.2 Materials

A 2-back task (Kirchner, 1958) was implemented in PsychoPy using custom Python scripts (Peirce et al., 2019). Participants were presented with a series of letters on a computer screen and instructed to press the spacebar when a letter matched the one presented two letters ago. The letter appeared for 500ms within a 2,000ms response window, before the next trial. Stimuli were pregenerated from a pool of letters, with a target frequency of 25% on average. Stimuli timing, target frequency and response window were determined based on pilot testing. A digital monitor (Dell, Optiplex 9010) with a screen resolution of 1280 x 1024 pixels and a refresh rate of 60Hz was used to display the task.

The IMI (Ryan et al., 1983) was used as the subjective IM measure. This 7-point Likert scale consists of 45 items spread across 7 subscales (from 1 = “not at all true” to 7 = “very true”). Three subscales were used for this study: Interest/enjoyment (7 items), perceived competence (6 items) and effort/importance (5 items). The IMI was selected for this study because it is considered a highly comprehensive and stable measure of IM, specific to participants' subjective experience of a laboratory experimental task. Specifically, it has been used to assess motivation and cognitive task performance (Cagna et al., 2024) and has shown good reliability (ICC = 0.70; Tsigilis and Theodosiou, 2003) and strong internal consistency for the n-back (α = 0.93; Ramme et al., 2022).

The Questionnaire for Eudaimonic WellBeing (QEWB) (Waterman et al., 2010) was used to measure the eudaimonic aspects of the participants' wellbeing. The questionnaire contains 21 items, to be answered using a 5-point Likert scale from “0-Strongly Disagree” to “4-Strongly Agree.” This scale has been proven to have strong internal consistency (α = 0.87), convergent validity and discriminant validity (Waterman et al., 2010; Schutte et al., 2013).

### 2.3 Procedure

In a single experimental session, participants were first provided with an information sheet outlining the study and their incentive of fixed credit or payment. After providing written consent, they completed an online questionnaire via Qualtrics (Qualtrics, 2024), stating basic demographic information (age, gender, year of study, dominant hand, whether they had a diagnosis of epilepsy and less than 5 h of sleep), reported any medical conditions that could affect performance and completed the QEWB. Participants were then given verbal instructions for the n-back task, after which they completed a practice block of 50 trials. If participants' accuracy did not exceed 75%, the practice block was repeated with new stimuli. The participants then proceeded with the main task, consisting of 5 blocks of 100 trials per block, with short self-paced breaks between blocks. Upon completion of the n-back, participants rated their motivation using the IMI. Participants were then given a free-choice option to voluntarily continue the n-back up to 3 additional blocks of 100 trials, stopping at any time, or instead, end the experiment straight away. Regarding elective blocks, fully scripted instructions were provided to standardize the procedure. Upon completion, participants were thanked for their participation, provided a debrief form, and compensated accordingly. The total experimental duration lasted 1 h.

### 2.4 Design and analysis

The present study employed a cross-sectional, between-groups, quantitative design. N-back performance was operationalised by accuracy, the proportion of correct responses, and RT to correct responses. To address the first and second hypothesis, a multiple linear regression was conducted with n-back task accuracy as the outcome variable and IM, year group and their interaction as predictors: *lm(accuracy* ~ *motivation* + *year* + *motivation: year)*. Additionally, a multiple linear regression was conducted to test RT as the outcome variable and IM, year group and their interaction as predictors: *lm(reaction time* ~ *motivation* + *year* + *motivation: year)*. The Inverse Efficiency Score (IES) was computed for each participant by dividing each participant's mean RT to correct responses by their proportion of correct responses (Statsenko et al., 2020):

$$\begin{array}{l} IES=\;\frac{mean\;correct\;RT}{proportion\;correct} \end{array}$$

A multiple linear regression was run to test the IES as the outcome variable and IM, year group and their interaction as predictors: *lm(IES* ~ *motivation* + *year* + *motivation: year)*. To address the third hypothesis, a simple linear regression was conducted with IM as the outcome variable and eudaimonic wellbeing as predictor: *lm(motivation* ~ *eudaimonic wellbeing)*.

## 3 Results

All data wrangling, analysis and visualization were conducted using R programming software (Version 4.5.2, R Core Team, 2025) through RStudio (v.1.1.456), using packages “tidyverse” (v2.0.0; Wickham et al., 2019), “psych“ (v2.6.1; Revelle, 2025), “car“ (v3.3; Fox et al., 2026), (v0.6.5; Garnier et al., 2023) and ‘pwr'(v1.3.0; Champely et al., 2020).

Total accuracy (number of correct responses) on the n-back was calculated for each participant. While RTs only showed slightly elevated levels of skewness (1.35), their kurtosis was high (5.48). Participants' RTs for correct responses on the n-back were initially trimmed ± 2 SD from the group mean to reduce the influence of extreme values and ensure that RT estimates reflected meaningful task engagement rather than lapses of attention (Berger and Kiefer, 2021). Following trimming, RT distributions showed minimal skewness (0.57) and acceptable kurtosis (2.67), indicating that the corrected kurtosis fell close to the value of 3 expected under normality. Participants' mean RTs were then calculated. Mean RT to correct responses was 632.20ms (*SD* = 98.98ms) and mean accuracy was 94.08% (*SD* = 2.98%).

IMI items were reverse-coded where necessary. Each participant's IMI total score was calculated by averaging responses across the 18 items. Descriptive statistics revealed that participants' mean IMI score was 4.38 (*SD* = 0.77). Cronbach's Alpha was calculated for the IMI, demonstrating good internal consistency (α = 0.86), indicating that the subscales used in this study were reliable (Tavakol and Dennick, 2011). The total QEWB score was calculated by adding up the 21 items, showing the mean total score of 61.43 (SD = 9.41) and a good internal consistency (α = 0.80).

### 3.1 Modeling performance

The main analysis modeled whether IM and task performance differed between first-and final-year undergraduate students. Rather than testing a simple association, the analysis specifically examined the interaction between IM and year group as we considered these factors to be closely related. Accordingly, a multiple linear regression model was specified to directly examine this interaction effect. The assumptions of linearity (visual inspection of a residual vs. fitted plot), normality (visual inspection of a quantile-quantile plot), and homoscedasticity were found to hold. Each predictor variable produced a value under five for the Variance Inflation Factor (VIF) tests, indicating no concern for multicollinearity (*VIF* = 2.03 for IMI scores, *VIF* = 1.00 for year group). Independence of residuals was confirmed using the Durbin-Watson Test (*D-W* = 2.08, *p* = 0.89).

Results from the regression revealed that the relationship between IM and accuracy did not significantly differ by year of study (*R*^2^ = 0.08, *F*_(3, 34)_ = 0.987, *p* = 0.41). The model explained only a small proportion of variance in accuracy, and the adjusted R^2^ value (*R*^2^ = −0.001) indicated negligible explanatory power after accounting for the number of predictors. Cohen's *f*^2^ indicated a small effect size (*f*^2^ = 0.087; Cohen, 1988). Ultimately, the model did not provide any evidence of a relationship between accuracy, IM and year group when considered together.

The regression model was also non-statistically significant for RT (*R*^2^ = 0.17, *F*_(3, 34)_ = 2.29, *p* = 0.096). However, this model accounted for a greater proportion of variance in RT than the accuracy model (Adjusted *R*^2^ = 0.09). Remarkably, matching IM scores were observed in first year (mean = 4.38, *SD* = 0.78) and final year students (mean = 4.38, *SD* = 0.79).

Although the models used to test the relationship were non-significant, it is likely that an attempt to include the additional factor of year group may have blurred the main effects of motivation. The multiple linear regression including IM, year group and their interaction as predictors was compared with a simpler model specifically testing the effect of IM on each respective performance metric in a simple linear regression. In fact, an analysis of variance showed that the larger multiple linear regression and a simple linear regression only including IM as predictor did not differ *F*_(3, 34)_ = 1.20, *p* = 0.315, suggesting that a simpler model could be used. Hence, the simpler linear regression confirmed a relationship of IM to reaction times, (*R*^2^ = 0.085, *F*_(1, 36)_ = 4.432, *p* = 0.042), showing lower RTs corresponding to higher IM levels. A comparison of models also indicated that the factor of year did not add to a model explaining accuracy (*F*_(3, 34)_ = 0.160, *p* = 0.853). However, this time the simple linear regression marginally missed significance at a uni-directional threshold (alpha = 0.1), (*R*^2^ = 0.046, *F*_(1, 36)_ = 2.77, *p* = 0.105), implying no effect of IM on accuracy.

**Figure 1:**
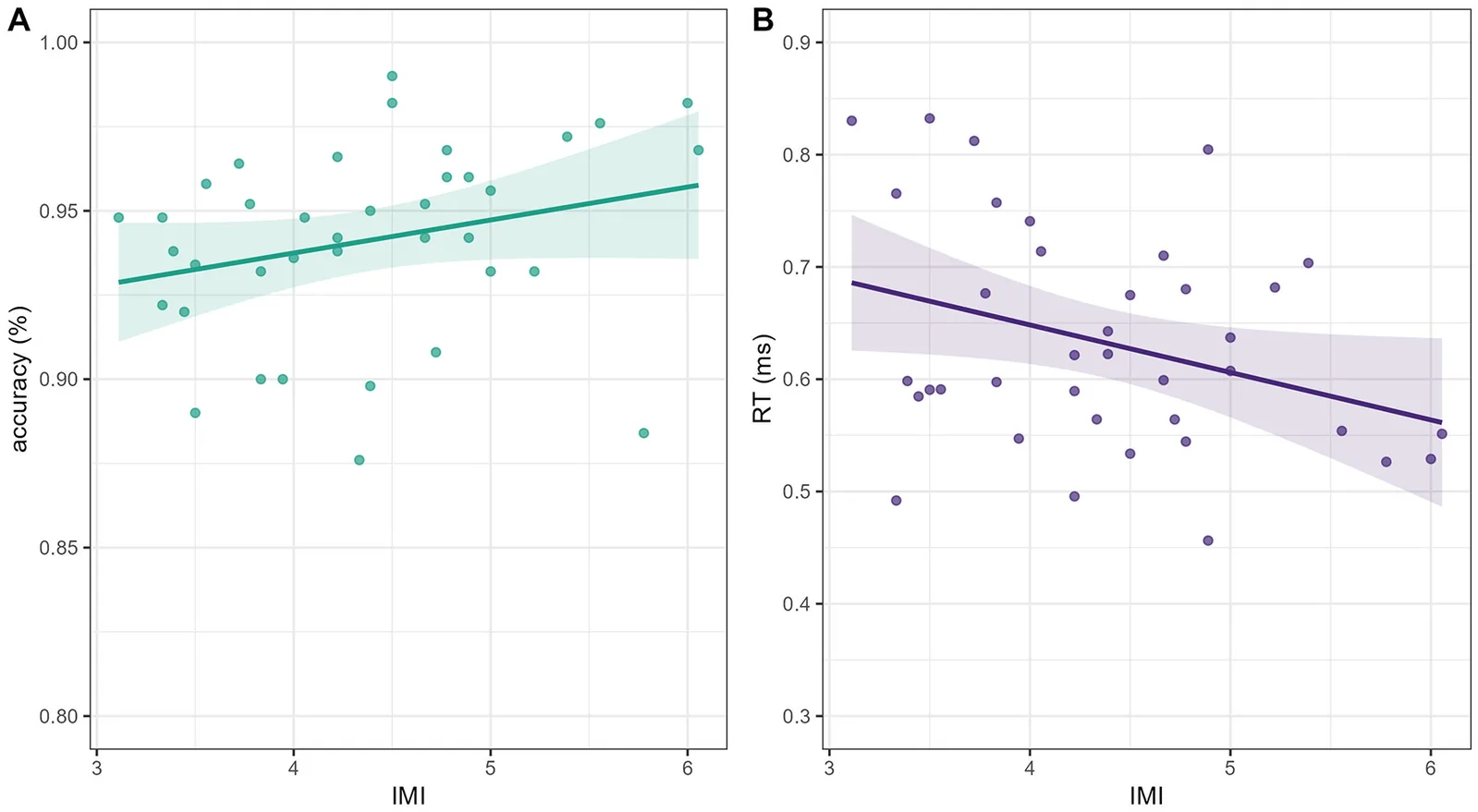
IMI scores and n-back performance. Participant IMI average scores and **(A)** accuracy (proportion of correct responses) and **(B)** RT (seconds). Each dot represents an individual participant. The solid lines represent the fitted linear regression slopes. The shaded areas indicate 95% confidence intervals around the regression lines.

This inconsistency when trying to characterize performance by reaction times and accuracy separately may reflect the fact they should be considered in alignment, especially considering an overall inversion of the slopes of the association of IMI to accuracy (Figure 1A) and RTs (Figure 1B). These could be identified as representing a speed-accuracy trade-off and provide further information about participant performance. Therefore, a further analysis was conducted using an inverse efficiency score (IES), integrating response speed and accuracy into a single performance metric (Statsenko et al., 2020). This enabled further examination of whether year-of-study effects emerged when performance was operationalised more holistically and to clarify the contribution of accuracy to performance. A multiple linear regression was conducted with IES as the outcome variable and IM, year group and their interaction as predictors. Assumptions of linearity, normality of residuals and homoscedasticity were assessed through visual inspection of diagnostic plots and were found to be adequately met. Independence of observations was assumed, as each participant contributed a single data point and the study employed a cross-sectional between-subject design with no repeated measures or temporal structure. Multicollinearity was confirmed by VIF (*VIF* = 2.03 for IMI scores, *VIF* = 1.00 for year group). IES values were also screened for distributional properties using boxplots and histograms to ensure suitability for regression analysis.

**Figure 2:**
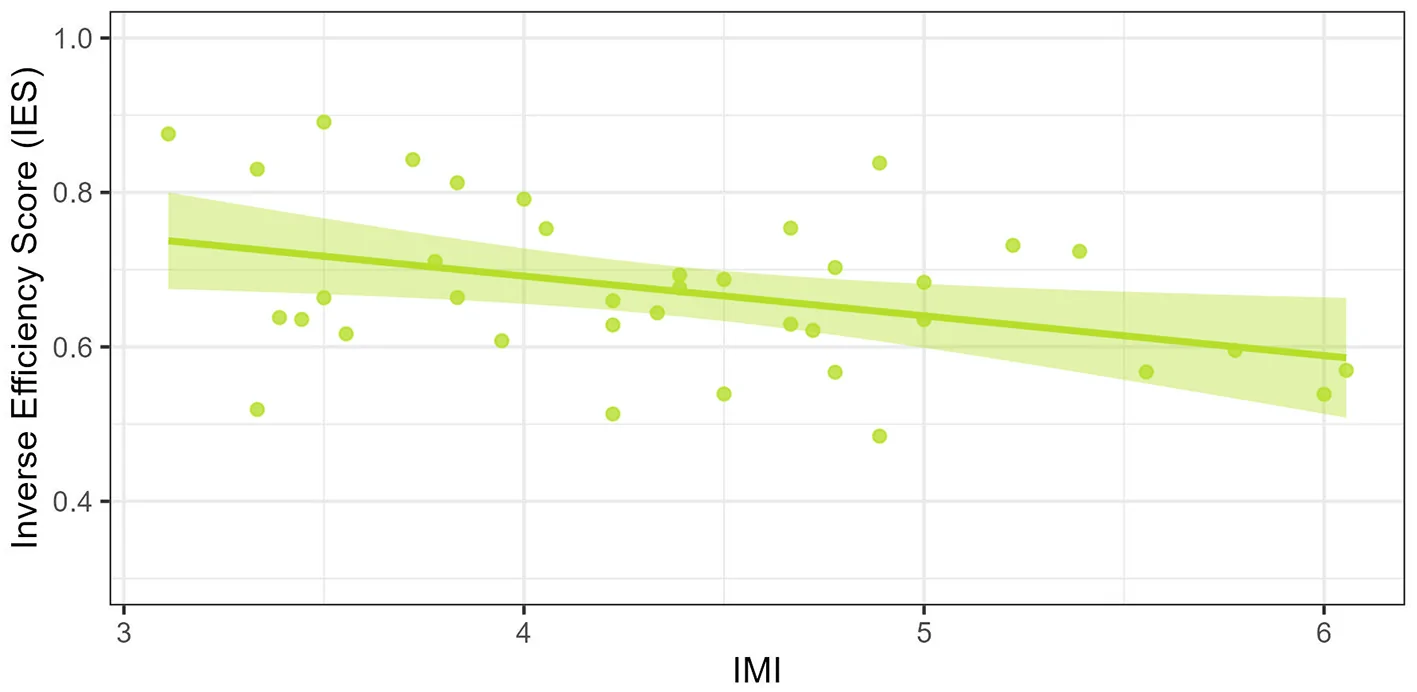
The relationship between IMI scores and IES scores. Each dot represents an individual participant. The solid lines represent the fitted linear regression slopes. The shaded areas indicate 95% confidence intervals around the regression lines. Lower IES values reflect greater task efficiency.

Results revealed that the model was not a significant predictor of inverse efficiency (*R*^2^ = 0.195, *F*_(3, 34)_ = 2.74, *p* = 0.058), explaining 19.5% of the variance in performance efficiency. Comparing the model with a simple linear regression predicting IES from IM, the removal of year group did not affect the model (*F*_(3, 34)_ = 1.32, *p* = 0.281). The simple linear regression predicting IES from IM was significant, showing that higher motivation led to higher efficiency in response (*R*^2^ = 0.094, *F*_(1, 36)_ = 4.84, *p* = 0.034). The negative relationship shows how much reaction time is required for one correct response, with lower IES indicating higher efficiency, as depicted in Figure 2.

### 3.2 Underlying factors in IM

In alignment with the pre-registered hypothesis, a simple linear regression tested whether eudaimonic wellbeing predicted participants' score on the IMI after completion of the n-back paradigm. Assumptions for linearity, normality (*p* = 0.53) and homoscedasticity (*p* = 0.32) were all met prior to analysis. The regression model was not significant, *F*_(1, 36)_ = 0.005, *p* = 0.940, and explained minimal variance of the IMI scores (${R^{2}}_{\text{adj}}$ = −0.028), suggesting that students' eudaimonic wellbeing was not predictive of their IM.

An additional exploratory analysis investigated the relationship between the IMI score and the number of free choice trials completed. This was done to test a prospective link of FCM to IM as an additional measure of participants' motivation within the task. The goal of the FCM was to capture motivation based on the number of trials they chose to complete at this optional stage. To examine whether students who persisted in voluntary engagement demonstrated higher IM than those who disengaged earlier, an exploratory analysis focused on the duration of voluntary participation on the FCM. Out of the 38 participants, 18 participants attempted the FCM, whilst 20 chose not to. Behavioral persistence was operationalised as the completion of the maximum possible number of free-choice trials (300 trials). Due to a non-normal distribution, a Spearman's correlation assessed the relationship between the two variables, finding a non-significant relationship *R*(35) = 0.132, *p* = 0.437. This suggests that the IMI and FCM as measures of IM were not related. Nevertheless, exploratory linear regressions were run to test a prospective relationship between performance metrics and voluntary time on task. The resulting models were not significant for any of the performance metrics: reaction times (*F*_(1, 36)_ = 0.082, *p* = 0.410), accuracy (*F*_(1, 36)_ = 0.025, *p* = 0.776) or IES (*F*_(1, 36)_ = 0.157, *p* = 0.695). Interestingly, participants who persisted to the maximum number of trials (*n* = 4) reported slightly higher IMI scores (*M* = 4.56; *SD* = 0.49) than participants who disengaged early (*M* = 4.30; *SD* = 0.74), although substantial overlap was observed. These findings suggest that, although in an anecdotal manner, participants completing all trials may prospectively comprise a motivationally distinct group.

## 4 Discussion

We investigated whether IM predicted cognitive task performance on the n-back and sought to underpin the difference with possible fluctuations of motivational levels between first- and final-year undergraduate students. Results revealed that higher IM was significantly associated with quicker reaction times. In addition, a measure of inverse efficiency capturing the speed-accuracy trade-off between reaction times and accuracy further showed higher efficiency in response to the task. In turn, the findings show consistently across several performance indicators that students with higher IM achieve better performance on the n-back. Contrary to expectations, year of study serving as a between-subject manipulation of IM did not produce a main effect or an interaction effect between IM and performance, underscoring that the present method of sampling from different academic years did not introduce the expected variability in task-elicited motivational states. Equally, the study found no link between eudaimonic wellbeing and IM. Overall, while the present study confirmed variability in student motivational states with an imprint on performance, it failed to identify any IM drivers. Collectively, the findings show that an unaccounted variability in student motivational states, rooted in different baseline levels of IM, drives divergence in performance on the n-back.

In addition, a lack of any link between IM and voluntary participation in the exploratory analysis of FCM provided additional insight into the relative importance of FCM as a measure of IM in the context of studying motivation's impact on performance. In the context of an n-back task, a classical cognitive task employing working memory and sustained attention, our investigation confirms that IMI or other self-report indicators of motivation map more reliably onto student performance.

### 4.1 Motivation and performance

By capturing a link between motivational states and performance specifically in students, this study aligns with prior research linking self-reported IM to task engagement and performance (Cerasoli et al., 2014; Taylor et al., 2014; Otermans et al., 2025) and extends prior work measuring motivation as a factor in cognitive performance (Cerasoli et al., 2014). By illustrating the impact of IM in an objective working memory paradigm, the findings provide empirical support for the proposition that IM not only enhances subjective report of performance as previously seen (Afzal et al., 2010; Isiksal, 2010; Augustyniak et al., 2016), but also objective, cognitive performance under controlled laboratory conditions (Peiró et al., 2019). Our findings remain consistent with SDT, which posits that individuals with higher IM invest deeper cognitive processing, autonomous effort and attention (Ritchie and Tucker-Drob, 2018; Fong et al., 2023), mechanisms relevant to cognitive paradigms like the n-back, thereby improving performance (Ryan and Deci, 2000, 2020; Huang et al., 2025).

However, the FCM findings limit this connection. The FCM sought to provide further grounding in the mechanisms of self-determination by examining whether self-reported IM predicted voluntary task persistence. Although marginally higher IM scores were observed among participants who completed all trials, IM did not decisively influence voluntary participation. These findings thus contrast with expectations derived from SDT and theoretical claims stating that voluntary time spent on tasks reflects IM (Deci, 1971; Deci and Ryan, 1985). We speculate that in this instance, persistence may not correlate with self-reported IM, as repetitive tasks may require additional regulatory processes beyond IM, including sustained autonomous engagement, vigilant attentional control and resistance to boredom and mind-wandering (Langner and Eickhoff, 2013; Brosowsky et al., 2023; Zanesco et al., 2025). The absence of a connection to the FCM may point to the limited capture of intrinsic interest in the task. IM is sensitive to perceived relevance and alignment with personal goals (Hulleman et al., 2010). When tasks lack intrinsic interest and contextual meaning, the correlation between behavioral persistence, like the FCM, and subjective motivation may weaken (Deci et al., 1999). Under such conditions, persistence may reflect tolerance for repetition, cognitive endurance or goal pursuit rather than intrinsic interest *per se* (Moshontz and Hoyle, 2021).

### 4.2 Baseline motivation in students

Contrary to expectations, the relationship between IM and task performance did not significantly differ between first- and final-year students. The absence of an interaction effect contrasts with research demonstrating a motivational decline across university (Pan and Gauvain, 2012; Almulla et al., 2025). In the context of the present sampling approach, IM instead functioned as a general facilitator of cognitive engagement across varied student groups. This aligns more with Brose et al. (2012), who demonstrated that momentary motivational fluctuations still predicted n-back performance even when controlling for background characteristics. We thus propose that, overshadowing any year group effects, IM may have functioned primarily at the level of moment-to-moment attentional engagement rather than reflecting developmental progression across years of study. From this perspective, IM merely enhanced the efficient allocation of executive resources regardless of academic stage. It is possible that academic motivational development may predominantly occur within education-relevant contexts, where identity, competence and task value are more closely aligned (Vansteenkiste et al., 2018; Lo et al., 2022; Cao and Ma, 2024). This aligns with our failure to detect a link between eudaimonic wellbeing and motivation, whereas previous research shows that eudaimonic predictor variables strongly correlate with subjective measures of IM (Waterman and Schwartz, 2024). Similar findings showed direct links between eudaimonic happiness and performance in the workplace, which is expected to be facilitated by IM (Kożusznik et al., 2019; Peiró et al., 2019). However, a crucial factor that is present in these studies, which the current tightly controlled laboratory study lacked, is that the task was meaningful. Delle Fave et al. (2011) found that the strongest components of meaning in life are family, work, and interpersonal relations. Interestingly, education was categorized as least important. This would be consistent with the lack of developmental effects in the present sample. It could be argued that the n-back paradigm falls into the education category (Beanland et al., 2020) for the tested students, as it involves testing, memory, and performance components. Since eudaimonic wellbeing is closely related to meaning within a task, the low-stakes aspects of the n-back may not have motivated the students (Penk et al., 2014).

As a possible implication of the present study, we offer a reflection on its connection to the current structural and academic pressures within the higher education system, including financial strains, reduced staff-student contact and support along with increased academic performance demands. It is possible that these recent pressures constrain opportunities for generalization of baseline IM differences in experimental task engagement (Hackley, 2025; Jones and Bell, 2025). We see the presently detected IM as operating at the level of cognitive engagement in students, while reflecting a general lack of meaning and fulfillment found in experimental participation. The lack of differentiation between first- and final-year students in both the main task performance and discretionary engagement on the FCM further suggests that the academic stage did not significantly alter IM expression in this sample. The discrepancy between behavioral engagement and the limited evidence of IM suggests that students may continue to participate cognitively while simultaneously experiencing reduced personal meaning or fulfillment. Qualitative studies likewise highlight a motivational need in students, rooted in concerns around performance (Blindheim et al., 2025). In fact, these have been noted to have real-world negative impacts, including increases in dropout rates (Zhang et al., 2025; Kovan, 2026). The findings thus help to illustrate the need to more carefully consider the self-reported element of motivation and its possible impact on student motivation broadly. This may in turn provide a more nuanced understanding of student engagement, wellbeing and persistence, whilst also helping to identify early signs of motivational decline that may contribute to experimental and wider academic disengagement.

### 4.3 Limitations and future research

While this study provides novel insights into the relationship between IM and cognitive performance among first- and final-year students, some methodological considerations limit the scope of interpretation and inform future research directions.

Crucially, the absence of a significant interaction between year groups suggests that manipulating baseline levels of motivation by selection within student samples is not an effective approach to between-subject manipulation of IM in a university student sample. Furthermore, a cognitive paradigm such as the n-back may have further elicited an IM that operates as a consistent facilitator of cognitive performance, rather than reflecting developmental progression. The choice of a 2-back task may have been insufficiently demanding for high-functioning university students, who typically demonstrate strong executive functioning, including WM capacity, cognitive control, and processing speed (Lawlor-Savage and Goghari, 2016; Guerra-Carrillo et al., 2017). In particular, sustained exposure to academic and social factors throughout university may strengthen fluid and crystallized cognitive abilities, potentially narrowing performance variance between year groups (Ritchie and Tucker-Drob, 2018; Borgeest et al., 2020). Future studies may benefit from employing higher-load paradigms, titrating task difficulty to individual performance levels, alternative approaches to RT analysis and enhancing sensitivity to differences in IM-related cognitive performance (Li et al., 2020). They may additionally consider how psychosocial factors shape performance in academic and experimental settings, as these were not measured in the present design. Previous work indicates that stereotype threat can reduce working memory capacity (Schmader and Johns, 2003), with comparable effects reported on the Stroop task (Hutchison et al., 2013). Because the n-back task requires participants to demonstrate cognitive ability under evaluative conditions resembling those of academic tests, stereotype threat may have influenced motivation as well as performance in the present study (Thoman et al., 2013). Future designs should therefore record the social identity variables relevant to stereotype threat and consider including a measure of perceived evaluative threat.

More generally, the present sample, drawn from UK university undergraduate psychology students, is considered higher in socioeconomic status than the UK population average (Palmer et al., 2021) and may thus present untracked confounds. We specifically note that the studied year groups may differ in their socioeconomic status, as students from lower socioeconomic backgrounds have higher attrition rates (Crawford, 2014). This attrition may thus lead to a more advantaged later year groups and result in an unexpected confound. Furthermore, research on the employed measure of eudaimonic wellbeing, the QEWB, has shown a need for further cross-cultural validation beyond Western samples (Church et al., 2014). Its validity may therefore be reduced for non-Western participants in the present sample. To better address these concerns, future related research should record socioeconomic and cultural background information.

Overall, our findings emphasize that self-report measures of IM are robustly reflected in task performance, whereas the absence of effects in FCM hints that additional motivational differences may emerge more clearly in discipline-relevant contexts (Breen and Lindsay, 2002), where autonomy and identity investment play a more decisive role. Future research could enhance ecological validity by embedding the n-back within academically meaningful contexts, for example, by incorporating academically relevant content or course-related stimuli. In particular, future designs should consider mastery goals, with focus on personal learning and development, as these are aspects of eudaimonic wellbeing which have been found to be strong predictors of IM (Bieg et al., 2017). These goals require inferences on personal competence and achievement by performing at one's best. As participants received no feedback on their performance throughout the completion of the n-back, they may have found it difficult to perform the best they could without any knowledge of their results or progress. Therefore, the task may not have tapped into the mastery goals element of eudaimonic wellbeing which relates to IM. It should be noted that Bieg et al. (2017) found no inverse relationship between IM and mastery goals, which explains why the present findings still related IM to performance.

### 4.4 Conclusion

In conclusion, this study extends prior research grounded primarily in self-reports and academic outcomes by demonstrating that IM is linked to objective cognitive task performance. Although this relationship did not significantly differ between first- and final-year students, the studied sample still presented with varied baseline levels of IM, enabling a demonstration of how IM consistently facilitates cognitive engagement across undergraduate stages. By integrating behavioral performance indices with motivational assessment, this research provides a more nuanced framework for examining how motivational processes translate into real-time cognitive performance. However, the absence of significant year-group and wellbeing effects suggests that the sources of this motivational variability remain to be identified, pointing to the need for designs that more directly manipulate or measure the conditions under which baseline IM differences emerge. The results highlight implications for higher education, emphasizing the importance of probing students' IM, particularly in the context of experimental participation, to enhance cognitive performance.

## Data Availability

The datasets presented in this study can be found in online repositories. The names of the repository/repositories and accession number(s) can be found below: https://osf.io/h9yrj?view_only=9c53a2cf0fd5454fba59bba9ba0587da.
